# 548. Biodegradable Synthetic Dermal Substitute for Conflict Area Burns and Trauma Patients

**DOI:** 10.1093/jbcr/irag033.245

**Published:** 2026-04-06

**Authors:** Noa Sabag, Gefen Bloom, Ofir Ron, Subhi Hakrush, Roy Grinberg, Eliran Jacobi, Inbal Alkalay, Dor Halpern, Eldad Silberstein, Yaron Shoham

**Affiliations:** Soroka University Medical Center, Beer Sheva, HaDarom; Soroka University Medical Center, Beer Sheva, HaDarom; Soroka University Medical Center, Beer Sheva, HaDarom; Soroka University Medical Center, Beer Sheva, HaDarom; Soroka University Medical Center, Beer Sheva, HaDarom; Soroka University Medical Center, Beer Sheva, HaDarom; Soroka University Medical Center, Beer Sheva, HaDarom; Soroka University Medical Center, Beer Sheva, HaDarom; Soroka University Medical Center, Beer Sheva, HaDarom; Soroka University Medical Center, Beer Sheva, HaDarom

## Abstract

**Introduction:**

During the past years our medical center dealt with a multitude of severe burns and complex multi-trauma patients as a result of the ongoing conflict in our region. Many of these patients suffered extensive soft tissue injuries without the ability to perform immediate wound closure or complex reconstruction due to their general condition, wound status, or lack of prolonged operating room availability due to mass casualty conditions. In such cases a temporary measure for wound bed coverage and preparation for closure was needed. The aim of this abstract is to report our experience using a biodegradable synthetic dermal substitute in such cases.

**Methods:**

This is an ongoing retrospective data collection study. Eligibility criteria included patients of all ages treated with biodegradable synthetic dermal substitute since December 2023. Patients underwent initial eschar removal or surgical debridement procedures until achieving a viable wound bed. The biodegradable synthetic dermal substitute was then applied. The dermal substitute is composed of a polyurethane open cell foam, which allows for neo-dermis formation, and is covered by a sealed silicone layer that maintains a clean wound environment until full integration. Split thickness skin graft coverage was then performed.

**Results:**

Between December 1st 2023 and September 29th 2025 we treated 25 patients suffering from complex soft tissue injuries with biodegradable synthetic dermal substitute. Soft tissue defects were the consequence of large burns post eschar removal, blast injury and multi-trauma. Delamination was performed after an average of 3.5 weeks. Skin graft take was >95% in all cases. There were no cases of infection or matrix loss. Complete wound closure was achieved in an average of 2.5 weeks after skin grafting.

**Conclusions:**

In our experience the use of this biodegradable synthetic dermal substitute is an efficient temporizing measure for use in complex soft tissue defects including exposed deep structures such as bone and tendon. It also allowed us to effectively treat complex injuries in a staged manner.

**Applicability of Research to Practice:**

We found the biodegradable synthetic dermal substitute a very useful tool, especially during mass casualty situations where patients’ condition or operating room availability limited the possibility of performing complex reconstructions.

**Funding for the study:**

N/A.

